# Aptamer-based sandwich-type biosensors

**DOI:** 10.1186/s13036-017-0054-7

**Published:** 2017-03-13

**Authors:** Ho Bin Seo, Man Bock Gu

**Affiliations:** Department of Biotechnology, College of Life Sciences and Biotechnology, Korea University, Anam-dong, Seongbuk-Gu, Seoul, 136-713 Republic of Korea

**Keywords:** A pair of aptamer, Aptasensor, Sandwich-type biosensors, Sandwich-type aptasensor, Aptamer-antibody complex

## Abstract

Sandwich-type biosensor platforms have drawn lots of attentions due to its superior features, compared to other platforms, in terms of its stable and reproducible responses and easy enhancement in the detection sensitivity. The sandwich-type assays can be developed by utilizing a pair of receptors, which bind to the different sites of the same target. In this mini-review paper, the sandwich-type biosensors using either pairs of aptamers or aptamer-antibody pairs are reviewed in terms of its targets and platforms, the schematic designs, and their analytical performance.

## Background

The bioreceptors, important part of the biosensors, are known to afford a major function of the biosensors, selectivity and sensitivity. The antibody is one of the well know bioreceptors widely used for its high avidity, specificity, and diversely applicable feature [1]. Since the sandwich-based assays using secondary antibodies were established in a diagnostic field, along with their enhancements in the sensitivity and specificity, these improvements have led to the commercial success in many ELISA or lateral flow strip type kits [2]. However, these antibody-based biosensors have some weak points in using antibodies, such as high cost, instability, or limitation of target kinds.

Aptamers, which are oligonucleotide or peptide bioreceptors specifically binding to the target molecules, have been considered as an alternative to antibodies in compensating the antibody’s weakness [3, 4]. Aptamers are known to be less expensive in its development, more stable in the wide ranges of pH and temperatures and less limitations in their targets than antibodies [5]. Aptamers are screened from a random library via systematic evolution of ligands by exponential enrichment (SELEX), which have been studied extensively since 1990 [6].

Similar to antibody-based biosensors, single aptamer-based biosensors have also suffered from its weak sensitivities, and so resulted in being destined to be unrealized for field application or commercialization. To overcome this limitation, alternatively, sandwich-type biosensors using a pair of aptamer and antibody complex [7–10] have been developed. In an antibody-aptamer pair or vice versa, the capture aptamer was immobilized on the platform, and the secondary antibody functionalized with signaling moieties bound to the captured target for generating signals. These sandwich-type bioassays showed enhanced sensitivity and specificity, compared to what a single aptamer is used [11].

Regarding the sandwich-type biosensors using a pair of aptamers, it has rarely been reported, probably due to less availability of the dual aptamers or a pair of aptamers, even though the dual aptamer-based sandwich-type assays are expected to be realized earlier. However, after the development of aptamer pairs are reported, a few sandwich-type biosensor applications using aptamer pairs have been continuously reported [12–15]. In other words, the successful development of aptamer pairs leads to the research on the development of appropriate sandwich-type biosensors for on-site diagnosis, similar to ELISA kits [16].

For the development of aptamer pairs, it is worth to mention new SELEX methods beyond traditional SELEX methods especially useful for developing a pair of aptamers or aptamer due, even though the SELEX itself is not the central issue in this review. The most conventional SELEX methods were based on the target immobilization, in which aptamer candidates can access to the targets where the surface of the targets is not occupied from immobilization [17]. Since the aptamer pairs have to bind the same target at different sites, an immobilization-free method should have advantages in the screening of aptamer pairs. For example, Graphene-Oxide SELEX (GO-SELEX), target immobilization-free SELEX, is one of the SELEX methods for developing aptamer pairs successfully [18]. GO-SELEX is based on a phenomenon that single strand DNAs adsorbed to graphene oxide surface strongly by **π**-**π** stacking. Successful screening of aptamer pairs for one protein target and two kinds of virus targets were reported based on the use of GO-SELEX so far [12–14]. Another SELEX method for aptamer pair is multivalent aptamer isolation SELEX (MAI-SELEX) [19]. MAI-SELEX has two distinct selection stages. The affinity module enriches for binding with a target on single binding site. The specificity module can separate the aptamer candidates into groups based on the binding sites. However, this method has the limitation of a target. The target should be separated into subunits for specificity module.

**Fig. 1:**
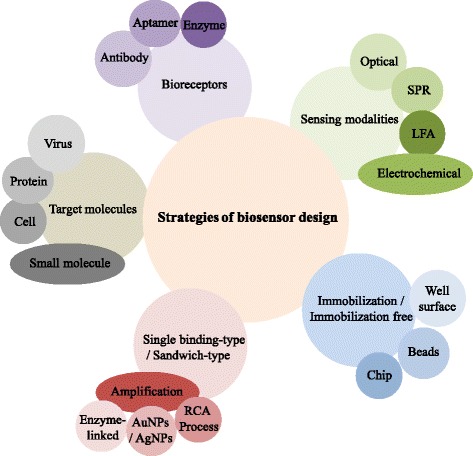
An illustration showing the parameters and factors to design biosensors strategically

In this review paper, the sandwich-type biosensors using pairs of aptamers or aptamer-antibody pairs are discussed regarding its targets and platforms, the schematic designs, and their analytical performance. The aptamer-based sandwich-type biosensors have been developed on various platforms such as electrochemical, localized surface plasmon resonance (LSPR), surface plasmon resonance (SPR), enzyme-linked aptamer-antibody sandwich (ELAAS), optical, or colorimetric-based platforms. Each platform has pros and cons, but there are advantages in common for the direction of on-site diagnosis by using a sandwich platform. These factors were summarized and illustrated as parameters and issues which should be considered to design biosensors strategically in the Fig. 1.

## The Sandwich-type biosensors using a pair of aptamer and antibody

**Fig. 2:**
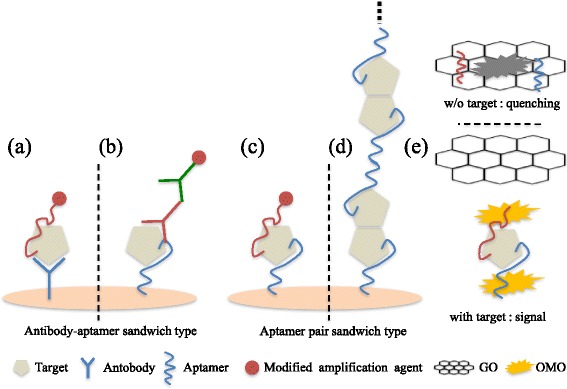
The scheme of aptamer-antibody-based sandwich-type biosensors and aptamer pair-based sandwich-type biosensors; Antibody-aptamer-based sandwich-type biosensor (**a**), aptamer-antibody-based sandwich-type biosensor (**b**), aptamer pair-based sandwich-type biosensor (**c**), aptamer-based sandwich-type biosensor using hands in hands nanostructure (**d**), FRET-based label free sandwich type biosensors (**e**)

When the secondary aptamers are not available, an alternative to aptamer pairs could be a pair of antibody and aptamer. In this case, the capture aptamers (or antibody but mostly not, since aptamers are not developed if the antibodies are available) are immobilized on the sensing platforms, and the secondary antibodies (or aptamers) are used for signal amplification or generation (Fig. 2 (a), (b)). There have been a few reports using a pair of both aptamer and antibody in the number of different platforms, such as electrochemical, LSPR, or optical-based sensors for the detection of protein or virus targets.

There is a trend toward developing platforms for on-site diagnosis. The electrochemical biosensors can be developed using miniaturized measuring device and platforms in a portable sensing platform, like a glucose meter. The reusable feature and small sample volume required for analysis are the advantages of LSPR biosensors. The ELAAS and optical-based biosensor, alternatives to ELISA-based biosensors, can enhance the sensitivity and selectivity in the detection of targets.

R-Q. Yu’s group developed a sandwich-type electrochemical sensor using an antibody and aptamer pair for the detection of thrombin [8]. The antibodies were immobilized on the electrode, and the extended aptamers were used as a secondary capture agent. They designed extended based in aptamer for the intercalation methylene blue into the extended and hybridized section as an electrochemical active indicator. This platform showed 0.5 nM of the limit of detection (LOD). L. Guo et al. developed an LSPR based biosensor induced by aptamer-antigen-antibody sandwich structures [7]. They used thrombin binding aptamer on Au nanorods as a capture receptor, and anti-thrombin antibody labeled as an LSPR signal amplification probe. This method enhanced LOD from 18.3 pM to 1.6 pM and makes the biosensor reusable.

Y. Huang et al. developed another sandwich-type electrochemical biosensor for the detection of Platelet-derived growth factor-BB (PDGF-BB) using a pair of antibody and aptamer [20]. The Rabbit anti-human PDGF-B polyclonal antibodies were immobilized on the electrode platform to capture PDGF-BB. Secondary primed-aptamers were used for the amplification of electrochemical signals by polymerizing circular DNA and methylene blue intercalated to the ds-DNA product. This sandwich polymerase amplification platform’s LOD is 18 pg/ml.

S. J. Lee et al. developed ELAAS assay for the detection of porcine reproductive and respiratory syndrome virus (PRRSV) type II [9]. They screened the PRRSV specific binding ss-DNA aptamer. This aptamer immobilized on the streptavidin-coated 96-well plate. The antibody and the HRP-conjugated antibody were used for the enzyme reaction-based signal generation on the sandwich format 96 well plates. The LOD of ELAAS system was 4.8 TCID_50_/ml. They claimed the ELAAS enhanced the LOD 5200-fold higher than SPR biosensor and PCR-based detection methods.

C. Preininger group presented an RNA aptamer and antibody-based biosensor for the detection of C-reactive protein (CRP), which has been identified as a biomarker for inflammation, sepsis and tissue necrosis [10]. The CRP binding aptamer was covalently immobilized on ARChip Epoxy. For the detection of bound CRP, dyes labeled secondary antibodies were used for generating optical signals in a sandwich format. They claimed that the aptamer-based biochip assay has more broad measuring range (10 μg/l to 100 mg/l) than antibody-based biochip assay for the diagnosis of low, elevated and high-risk patients.

By replacing one of the bioreceptors in the sandwich-type biosensors, i.e., the capture antibody or detection antibody with aptamer for high sensitivity and selectivity, researchers have suggested a new sandwich-type platforms using a pair of aptamer-antibody or vice versa. Limitations of using antibodies, such as high cost and instability, can be overcome by using aptamer pair-based sandwich type platforms in next subject.

## The Sandwich-type aptasensors using a pair of aptamers

The sandwich-type aptasensors using a pair of aptamers have been reported with their enhanced sensitivity and specificity in various platforms (Fig. 2 (c)). The different platforms using dual aptamers have been implemented in the number of different platforms, such as colorimetric, electrochemical, or SPR-type platforms. Each type of these platforms has advantages for signal amplification by using secondary aptamer and properly fitted for on-site diagnosis. In fact, the comparisons between the sandwich-type aptasensors and immunosensors were conducted and summarized briefly in the Table 1.

**Table 1:** A brief comparison between the sandwich-type aptasensors and immunosensors in terms of sensing modalities, sensing platforms, and sample matrices

Target	Bioreceptors	Sensing modalities	Sensing platform	Sample matrices	LOD	Reference
Thrombin	Ab + Apt	Electrochemical sandwich	On the electrode	Tris-HCl buffer	0.5 nM	[8]
	Ab + Apt	LSPR sandwich	Solution phase	Binding buffer/male human serum	1.6 pM/50 pM	[7]
	Apt pair	Colorimetric sandwich	96 well plate	Binding buffer	0.4 μM	[16]
	Ab	Impedance	On the electrode	PBS	100 nM	[23]
	Apt pair	Impedance	On the electrode	Binding buffer	100 nM	[23]
	Apt pair	FRET label free	Solution phase	Tris-HCl buffer/Serum	0.76 nM/130 nM	[24]
PDGF-BB	Ab + Apt	Electrochemical sandwich	On the electrode	Tris-HCl buffer/Human serum	18 pg/ml	[20]
	Apt pair	Electrochemical sandwich	On the electrode	Purified sample/Blood serum	10 fM/1 pM	[29]
	Apt pair	Electrochemical multiple sandwich	On the electrode	Tris-HCl buffer	Less than 100fM	[28]
	Ab	Electrochemical sandwich	On the electrode	Human serum	0.01 ng/ml	[35]
CRP	Ab + Apt	Optical sandwich assay	On AR chip epoxy	Human serum	10 μg/l	[10]
	Ab pair	Electrochemical snadwich	Electrode array	Human serum	15 fg/ml	[36]
Ramos cell	Apt pair	LFA chip	Nitrocellulose membrane strip	Human blood	800 cells	[25]
MCF-7	Apt pair	Electrochemical sandwich	On the electrode	PBS	100 cells	[26]
	Ab pair	Electrochemiluminescence	on the electrode	Human serum	4.5 fg/ml	[37]
*S. aureus*	Apt pair	Electrochemical sandwich	Solution phase	Tris-HCl buffer/real tap water and river sample	1.0 CFU/ml/134.3 CFU/ml	[30]
	Ab	Electrochemical sandwich	On the electrode	Phospate buffer	1000 cells/ml	[38]
VEGF	Apt pair	SPR sandwich	on the Au chip	Tris-HCl buffer	100 pg/ml	[31]
	Ab pari	Electrochemical sandwich	On the electrode	Human serum	21 cells/ml	[39]
Vaspin	Apt pair	SPR sandwich	on the Au chip	Binding buffer/Human serum	3.5 ng/ml/4.7 ng/ml	[14]
	Ab + Apt	SPR based ELAAS	on the Au chip	Human serum	39 ng/ml	[11]
PRRSV type II	Ab + Apt	ELAAS	96 well plate	Binding buffer/Swine serum	4.8 TCID50/ml	[9]
PRRSV	Ab	Electrochemical sandwich	On the electrode	Negative serum	380 pg/ml	[40]
BVDV	Apt pair	SPR sandwich	on the Au chip	Binding buffer	500 TCID50/mL	[13]
	Ab	Light scattrering	Micro fluidic cahnnel	Tissue culture media and fetal calf serum	10 TCID50/mL	[41]
H5N1	Apt pair	SPR sandwich	on the Au chip	Binding buffer	200 EID50/ml	[12]
	Ab	Electrochemical sandwich	Micro fluidic cahnnel	Buffer	1 pg/ml	[42]

The colorimetric biosensor can be widely used for many cases because the result of a colorimetric biosensor can be analyzed by bare eye roughly without measuring device. The SPR-based biosensor needs SPR device for analysis, but known to have high sensitivity. So this biosensor can be a better choice for very diluted samples. Each aptasensor platform using a pair of aptamers and its analytical performances were summarized below.

Thrombin is a coagulation protease generated at sites of vascular injury. This protein activates platelets, leukocytes and endothelial cells [21]. Thrombin has a role of a biomarker of a few diseases, including atherosclerosis and stroke [22]. W-Y. Chen’s group presented a sandwich-type colorimetric aptasensor using a pair of thrombin binding aptamers [16]. The capturing aptamer immobilized on an avidin, while the secondary aptamer conjugated with Pt for the reaction with TMB (3,3′,5,5′-tetramethylbenzidine). The limit of detection was 0.4 *μ*mol/L for this sandwich colorimetric aptasensor. U. Schecht et al. compared antibody and aptamer receptors for the detection of thrombin with a nanometer gap-sized impedance biosensor platform [23]. They immobilized antibodies and RNA-aptamers on two same biosensor platforms respectively. The result showed both antibody and aptamer equally suitable for the specific detection of thrombin. The aptamer-based biosensor made faster binding than the antibody-based biosensor. However, the antibody-based biosensor had a higher signal than the aptamer-based biosensor. In a low thrombin concentration, the aptamer-based sensor was found to be a little bit more sensitive than the antibody-based sensor. J. Li et al. developed label-free thrombin Fluorescence resonance energy transfer (FRET) aptasensors using [Ru(bpy)_2_(o-mopip)]^2+^ (OMO) and graphen oxide (GO) [24]. The OMO have an interaction with GO. When the target is induced, the aptamer and OMO detached out from GO for signal readouts (Fig. 2 (e)). The high sensitivity was obtained by using an aptamer pair in a sandwich complex formation (LOD; 0.76 nM).

G. Liu et al. presented nanoparticle strip aptasensor for detection of cancer cell (Ramos cell) using a pair of aptamers [25]. Aptamers selected by cell-SELEX and were prepared with gold nanoparticles (AuNPs) for visibility on lateral flow assay chip. Without any instrumentation, the LOD was 4000 Ramos cells, but with portable strip reader, they could detect 800 Ramos cells within 15 min. X. Zhu et al. represented a electrochemical aptasensor for the detection of Michigan cancer foundation-7 (MCF-7) human breast cancer cells [26]. A mucin 1 (overexpressed glycoprotein on apical surface of cancer cells) binding aptamer was used as capture and detection aptamer. Capture aptamer immobilized on a gold electrode and detection aptamer was labeled with HRP enzyme for electrochemical response catalysis. This method showed low LOD of 100 cells.

Platelet-derived growth factor (PDGF) is a potent mitogen protein for vascular smooth muscle cells implicated in the pathogenesis of atherosclerosis. A concentration-dependent contraction of aortic strips is caused by PDGF, which contributes to the increased vasoreactivity of atherosclerotic vessels [27]. C. Li et al. presented multiple sandwich-type electrochemical aptasensor for the detection of Platelet-derived growth factor-BB (PDGF-BB) [28]. The primary capturing aptamer was immobilized on a gold electrode, and two different kinds of secondary aptamers were used for multiple sandwich-type assays. Each secondary aptamer have complementary regions for hybridization. The third aptamer can capture another target proteins (Fig. 2 (d)). They also used redox species ([Ru(NH_3_)_6_]^3+^) for producing a remarkably amplified signal. This multiple sandwich-type aptasensor showed high sensitivity (LOD less than100 fM). J. Wang et al. reported sandwich-type electrochemical aptasensor for the detection of PDGF using AuNPs and redox species [29]. The detection sensitivity was enhanced by using signal amplification agents, AuNPs and [Ru(NH_3_)_5_Cl]^2+^. They showed extraordinarily low LOD of 10 fM for purified samples, 1 pM for contamicated-ridden samples or undiluted blood serum.

N. Soltani group developed electrochemical aptasensor for detection of *Staphylococcus aureus* (*S.aureus*) using aptamer-conjugated silver nanoparticles (AgNPs) [30]. A pairs of aptamers were conjugated to magnetic beads and AgNPs for anodic stripping voltammetry, respectively. This platform showed sensitive results in LOD 1.0 CFU/ml, and a similar performance in real water sample.

H. Chen et al. presented sandwich-type SPR based aptasensor using rolling circle amplification (RCA) process to amplify the SPR signal for the detection of vascular endothelial growth factor (VEGF) [31]. The capture aptamer immobilized on gold chip and carboxyl-coated polystyrene microspheres were used to load the detection aptamer and primer for RCA process. After the addition of ligase, phi29 polymerase, and dNTPs, the RCA performed SPR signal amplification. The detection limit of 100 pg/ml enhanced about 2-fold higher than the previous studies, electrochemical and optical sensing platforms [32, 33].

Visceral adipose tissue-derived serpin (vaspin) is identified as an adipokine known as a protein biomarker for insulin resistance involved in obesity and type-2 diabetes [34]. N. Hanun et al. presented a sandwich-type aptasensor for detection vaspin using aptamer pair [14]. As a first vaspin binding aptamer duo, this pair of aptamers was developed by immobilization-free GO-SELEX method. They characterized the candidates of aptamer duo by sandwich-type SPR based assay. Primary aptamer was immobilized on a gold chip, while the possible secondary aptamer was conjugated with AuNPs, which can make the amplification of SPR signals. This aptamer duo’s sandwich bindings were proved by using confocal laser scanning microscopy (CLSM) and circular dichroism (CD) analysis after quantum dot (QD)-labeled reporter aptamer used. This aptasensor’s limit of detection was 3.5 ng/ml, while the sensitivity was improved 114 times than aptasensor using a single aptamer.

J-W Park et al. developed sandwich-type aptasensor using a dual aptamer for the detection of bovine viral diarrhea virus (BVDV) [13]. They also screened a pair of aptamers by GO-SELEX for a whole virus. The SPR based sandwich assay was performed to obtain the aptamer pair’s affinity and specificity. This aptasensor showed 500 TCID_50_ mL − 1 limit of detection. The sensitivity was enhanced 20 fold than aptasensor using a single aptamer.

V-T. Nguyen et al. developed sandwich-type SPR based aptasensor for detection H5Nx viruses using a pair of aptamers [12]. The pairs of aptamers were screened by Multi-GO-SELEX with whole virus targets. Aptamers screened out by Multi-GO-SELEX have flexibility to binding multiple targets, such as H5N1and H5N2, H5N1 and H5N8 or H5N1, or H5N2 and H5N8. In addition, a pair of aptamers binding to different site of the same whole H5N1 virus was also screened out by this SELEX method. The limit of detection of this pair of aptamers is 200 EID_50_/ml in a sandwich-type SPR based aptasensor.

## Conclusion

The sandwich-type assay platform is essential to develop a highly sensitive biosensor. For the development of aptamer pair-based sandwich platforms, the successful screening of a pair of aptamers is required. The limitations of the sandwich-type biosensors using antibodies have been solved after the pairs of aptamers were developed. The development of sandwich-type aptasensors for rapid, sensitive, and competitive on-site diagnosis are expected to be continued by researchers with the development of new pairs of aptamers for various targets which could be done via easy, low cost, and convenient aptamer screening methodologies.

## Abbreviations

AgNPs: Silver nanoparticles
APT: Aptamer
AuNPs: Gold nanoparticles
BVDV: Bovine viral diarrhea virus
CD: Circular dichroism
CLSM: Confocal laser scanning microscopy
CRP: C-reactive protein
ELAAS: Enzyme-linked aptamer-antibody sandwich
GO: Graphene-Oxide
LOD: Limit of detection
LSPR: Localized surface plasmon resonance
MAI-SELEX: Multivalent aptamer isolation SELEX
MCF-7: Michigan cancer foundation-7
OMO: [Ru(bpy)_2_(o-mopip)]^2+^
PDGF-BB: Platelet-derived growth factor-BB
PRRSV: Porcine reproductive and respiratory syndrome virus
QD: Quantum dot
RCA: Rolling circle amplification
SELEX: Systematic evolution of ligands by exponential enrichment
SPR: Surface plasmon resonance
TMB: 3,3′,5,5′-tetramethylbenzidine
vaspin: Visceral adipose tissue-derived serpin
VEGF: Vascular endothelial growth factor

## References

[1] North JR (1985). Immunosensors - antibody-based biosensors. Trends Biotechnol.

[2] Pei XM, Zhang B, Tang J, Liu BQ, Lai WQ, Tang DP (2013). Sandwich-type immunosensors and immunoassays exploiting nanostructure labels: a review. Anal Chim Acta.

[3] Toh SY, Citartan M, Gopinath SCB, Tang TH (2015). Aptamers as a replacement for antibodies in enzyme-linked immunosorbent assay. Biosens Bioelectron.

[4] Cao Y, Li GX, Cai W (2014). Engineering aptamers for biomedical applications: part I. Engineering in translational medicine.

[5] Song SP, Wang LH, Li J, Zhao JL, Fan CH (2008). Aptamer-based biosensors. Trac-Trend Anal Chem.

[6] Tuerk C, Gold L (1990). Systematic evolution of ligands by exponential enrichment - Rna ligands to bacteriophage-T4 DNA-polymerase. Science.

[7] Guo LH, Kim DH (2012). LSPR biomolecular assay with high sensitivity induced by aptamer-antigen-antibody sandwich complex. Biosens Bioelectron.

[8] Kang Y, Feng KJ, Chen JW, Jiang JH, Shen GL, Yu RQ (2008). Electrochemical detection of thrombin by sandwich approach using antibody and aptamer. Bioelectrochemistry.

[9] Lee SJ, Kwon YS, Lee JE, Choi EJ, Lee CH, Song JY, Gu MB (2013). Detection of VR-2332 strain of porcine reproductive and respiratory syndrome virus type II using an aptamer-based sandwich-type assay. Anal Chem.

[10] Pultar J, Sauer U, Domnanich P, Preininger C (2009). Aptamer-antibody on-chip sandwich immunoassay for detection of CRP in spiked serum. Biosens Bioelectron.

[11] Lee SJ, Park JW, Kim IA, Youn BS, Gu MB (2012). Sensitive detection of adipokines for early diagnosis of type 2 diabetes using enzyme-linked antibody-aptamer sandwich (ELAAS) assays. Sensor Actuat B-Chem.

[12] Nguyen VT, Seo HB, Kim BC, Kim SK, Song CS, Gu MB (2016). Highly sensitive sandwich-type SPR based detection of whole H5Nx viruses using a pair of aptamers. Biosens Bioelectron.

[13] Park JW, Lee SJ, Choi EJ, Kim J, Song JY, Gu MB (2014). An ultra-sensitive detection of a whole virus using dual aptamers developed by immobilization-free screening. Biosens Bioelectron.

[14] Raston NHA, Gu MB (2015). Highly amplified detection of visceral adipose tissue-derived serpin (vaspin) using a cognate aptamer duo. Biosens Bioelectron.

[15] Xie SN, Walton SP (2010). Development of a dual-aptamer-based multiplex protein biosensor. Biosens Bioelectron.

[16] Higuchi A, Siao YD, Yang ST, Hsieh PV, Fukushima H, Chang Y, Ruaan RC, Chen WY (2008). Preparation of a DNA aptamer-Pt complex and its use in the colorimetric sensing of thrombin and anti-thrombin antibodies. Anal Chem.

[17] Stoltenburg R, Reinemann C, Strehlitz B (2007). SELEX-A (r)evolutionary method to generate high-affinity nucleic acid ligands. Biomol Eng.

[18] Park JW, Tatavarty R, Kim DW, Jung HT, Gu MB (2012). Immobilization-free screening of aptamers assisted by graphene oxide. Chem Commun.

[19] Gong Q, Wang JP, Ahmad KM, Csordas AT, Zhou JH, Nie J, Stewart R, Thomson JA, Rossi JJ, Soh HT (2012). Selection strategy to generate aptamer pairs that bind to distinct sites on protein targets. Anal Chem.

[20] Huang Y, Nie XM, Gan SL, Jiang JH, Shen GL, Yu RQ (2008). Electrochemical immunosensor of platelet-derived growth factor with aptamer-primed polymerase amplification. Anal Biochem.

[21] Connolly AJ, Ishihara H, Kahn ML, Farese RV, Coughlin SR (1996). Role of the thrombin receptor In development and evidence for a second receptor. Nature.

[22] Lin KY, Kwong GA, Warren AD, Wood DK, Bhatia SN (2013). Nanoparticles that sense thrombin activity as synthetic urinary biomarkers of thrombosis. ACS Nano.

[23] Schlecht U, Malave A, Gronewold T, Tewes M, Lohndorf M (2006). Comparison of antibody and aptamer receptors for the specific detection of thrombin with a nanometer gap-sized impedance biosensor. Anal Chim Acta.

[24] Li JJ, Hu XC, Shi S, Zhang YW, Yao TM (2016). Three label-free thrombin aptasensors based on aptamers and [Ru(bpy)(2)(o-mopip)](2+). J Mater Chem B.

[25] Liu GD, Mao X, Phillips JA, Xu H, Tan WH, Zeng LW (2009). Aptamer-nanoparticle strip biosensor for sensitive detection of cancer cells. Anal Chem.

[26] Zhu XL, Yang JH, Liu M, Wu Y, Shen ZM, Li GX (2013). Sensitive detection of human breast cancer cells based on aptamer-cell-aptamer sandwich architecture. Anal Chim Acta.

[27] Berk BC, Alexander RW, Brock TA, Gimbrone MA, Webb RC (1986). Vasoconstriction - a new activity for platelet-derived growth-factor. Science.

[28] Li C, Wang ZY, Gao T, Duan AP, Li GX (2013). Fabrication of hand-in-hand nanostructure for one-step protein detection. Chem Commun.

[29] Wang J, Meng WY, Zheng XF, Liu SL, Li GX (2009). Combination of aptamer with gold nanoparticles for electrochemical signal amplification: Application to sensitive detection of platelet-derived growth factor. Biosens Bioelectron.

[30] Abbaspour A, Norouz-Sarvestani F, Noon A, Soltani N (2015). Aptamer-conjugated silver nanoparticles for electrochemical dual-aptamer-based sandwich detection of staphylococcus aureus. Biosens Bioelectron.

[31] Chen HX, Hou YF, Qi FJ, Zhang JJ, Koh K, Shen ZM, Li GX (2014). Detection of vascular endothelial growth factor based on rolling circle amplification as a means of signal enhancement in surface plasmon resonance. Biosens Bioelectron.

[32] Freeman R, Girsh J, Jou AFJ, Ho JAA, Hug T, Dernedde J, Willner I (2012). Optical aptasensors for the analysis of the vascular endothelial growth factor (VEGF). Anal Chem.

[33] Zhao S, Yang WW, Lai RY (2011). A folding-based electrochemical aptasensor for detection of vascular endothelial growth factor in human whole blood. Biosens Bioelectron.

[34] Van Harmelen V, Reynisdottir S, Eriksson P, Thorne A, Hoffstedt J, Lonnqvist F, Arner P (1998). Leptin secretion from subcutaneous and visceral adipose tissue in women. Diabetes.

[35] Qu FL, Lu HM, Yang MH, Deng CY (2011). Electrochemical immunosensor based on electron transfer mediated by graphene oxide initiated silver enhancement. Biosens Bioelectron.

[36] Krause CE, Otieno BA, Bishop GW, Phadke G, Choquette L, Lalla RV, Peterson DE, Rusling JF (2015). Ultrasensitive microfluidic array for serum pro-inflammatory cytokines and C-reactive protein to assess oral mucositis risk in cancer patients. Anal Bioanal Chem.

[37] Wang JX, Zhuo Y, Zhou Y, Yuan R, Chai YQ (2015). Electrochemiluminescence immunosensor based on multifunctional luminol-capped AuNPs@Fe3O4 nanocomposite for the detection of mucin-1. Biosens Bioelectron.

[38] Rishpon J, Ivnitski D (1997). An amperometric enzyme-channeling immunosensor. Biosens Bioelectron.

[39] Tabrizi MA, Shamsipur M, Sarkar S, Zolfaghari N (2017). An ultrasensitive sandwich-type electrochemical immunosensor for the determination of SKBR-3 breast cancer cell using rGO-TPA/FeHCFnano labeled Anti-HCT as a signal tag. Sensor Actuat B-Chem.

[40] Shao K, Zhang C, Ye S, Cai K, Wu L, Wang B, Zou C, Lu Z, Han H (2017). Near–infrared electrochemiluminesence biosensor for high sensitive detection of porcine reproductive and respiratory syndrome virus based on cyclodextrin-grafted porous Au/PtAu nanotube. Sensor Actuat B-Chem.

[41] Heinze BC, Song JY, Lee CH, Najam A, Yoon JY (2009). Microfluidic immunosensor for rapid and sensitive detection of bovine viral diarrhea virus. Sensor Actuat B-Chem.

[42] Han JH, Lee D, Chew CHC, Kim T, Pak JJ (2016). A multi-virus detectable microfluidic electrochemical immunosensor for simultaneous detection of H1N1, H5N1, and H7N9 virus using ZnO nanorods for sensitivity enhancement. Sensor Actuat B-Chem.

